# Minimally Invasive Thoracic Surgery in Pediatric Patients: The Taiwan Experience

**DOI:** 10.1155/2013/850840

**Published:** 2013-05-30

**Authors:** Yu-Kai Huang, Chieh Chou, Chung-Liang Li, Hui-Gin Chiu, Yu-Tang Chang

**Affiliations:** ^1^Department of Surgery, Kaohsiung Medical University Hospital, 100 Tzyou 1st Road, Kaohsiung 80708, Taiwan; ^2^Department of Surgery, Faculty of Medical School, College of Medicine, Kaohsiung Medical University, 100 Shi-Chuan 1st Road, Kaohsiung 80708, Taiwan; ^3^Division of Pediatric Surgery, Department of Surgery, Kaohsiung Medical University Hospital, Kaohsiung Medical University, Kaohsiung 80708, Taiwan

## Abstract

Minimally invasive technology or laparoscopic surgery underwent a major breakthrough over the past two decades. The first experience of thoracoscopy in children was reported around 1980 for diagnosis of intrathoracic pathology and neoplasia. Up until the middle of the 1990s, the surgical community in Taiwan was still not well prepared to accept the coming era of minimally invasive surgery. In the beginning, laparoscopy was performed in only a few specialties and only relatively short or simple surgeries were considered. But now, the Taiwan's experiences over the several different clinical scenarios were dramatically increased. Therefore, we elaborated on the experience about pectus excavatum: Nuss procedure, primary spontaneous hemopneumothorax, thoracoscopic thymectomy, and empyema in Taiwan.

## 1. Introduction

Pediatric surgery is a specialized field of surgery that aims to correct congenital abnormalities in fetuses, infants, children, adolescents, or young adults. Pediatric surgeons use operative techniques from different surgical fields to correct different kinds of pediatric conditions that may involve more than one organ. These surgical fields can include, but are not limited to general surgery, neurological surgery, ophthalmology, otolaryngology, orthopedic surgery, plastic surgery, and urology. The range of skills required for problem solving may not be limited to specific fields, and cooperation with other specialties is almost always needed.

As we all know, one cannot view pediatric surgical patients as merely miniature version of adults. The physique, anatomy, pathophysiology, and course of diseases in this particular small group of patients are quite different from adults. Unique challenges and problems come from basic differences such as smaller size, relative immunodeficiency, and immature cardiopulmonary functions. Therefore, the surgical procedures, techniques, and postoperative care of children differ markedly from those of adults. For these reasons special consideration and care are needed not only to cope with disease-related distress, but also to fulfill demands of both aesthetics and functioning, which are likely the major concerns of pediatric surgeons. 

Difference in surgical approach may result in lifelong consequences in children undergoing major operative interventions. Pediatric surgeons always attempt to operate in the least invasive way, causing as minimal collateral injuries as possible. It seems that laparoscopic approach may be the perfect solution to this consideration. However, at the time when laparoscopic techniques were initially described and introduced in adult patients, its use and benefits in the pediatric age group were more questionable. The main reason for this situation would be the lag in development of pediatric laparoscopic equipment. Some landmark and well-performed laparoscopic procedures used in adults, such as surgical treatment for cholelithiasis, were uncommonly used in the pediatric age group, which also happens to have a much lower incidence rate. Low case volume implies less demand and profit, which diminishes the interest of equipment investors and causes manufacturing and development to fall behind. 

Laparoscopic approach to surgical treatment began in the early 20th century and minimally invasive technology or laparoscopic surgery underwent a major breakthrough over the past two decades. However, pediatric laparoscopy was still restricted mainly to diagnostic use for a long period. As for the pediatric therapeutic field, Stephen Gans reported in early 1973 the first evaluation of a contralateral patent processus vaginalis and the development of pediatric surgical instruments [1, 2]. Besides, Götz et al. first reported pediatric laparoscopic appendectomy in 1990 [3]. These pioneers contributed greatly to this field and the advance in laparoscopy apparatus made minimally invasive surgeries possible and more suitable for the pediatric population. In addition, newer technological improvements in life support, vital sign monitoring, and endotracheal anesthesia allowed for wider, safer, and more efficient application, particularly on pediatric patients. These developments also enabled pediatric surgeons to perform even the most complicated intra-abdominal or intrathoracic procedures through laparoscopy and thoracoscopy. 

The first experience of thoracoscopy in children was reported around 1980 for diagnosis of intrathoracic pathology and neoplasia [4, 5]. Technology and technique modified of thoracoscopy provide excellent access and visualization. Thoracoscopy was then being used extensively for lung biopsy and evaluating various intrathoracic lesions. Thoracoscopic visualization of the pleural cavity permits efficient debridement, thorough adhesiolysis, and optimal placement of drainage tubes in the treatment of empyema in children [6].

However, even though thoracoscopic approach has clearly shown significant benefits over standard open thoracotomy in many cases, there have been only small numbers of reports for thoracoscopy in small children and infants in early 1990s. Since the early 20th century, laparoscopic approach to surgical treatment has gained increasing popularity in adults. The result is an increasingly wider application to the use of thoracoscopy in children. Miniaturization of instrumentation and telescope improved optics such as high-resolution digital camera, and light source allows the surgeon to perform much finer movements. Extensive intrathoracic procedures can be performed in infants as small as 1 kg. There are also a number of energy sources such as Ligasure (Valleylab, Boulder, CO, USA) available to provide hemostasis, all of which have enabled pediatric surgeons to perform even the most complicated intrathoracic procedure thoracoscopically. Currently, video-assisted thoracoscopy (VATS) has become an increasingly important tool in pediatric surgery. It has been mainly used for lung biopsy and for the treatment of empyema and recurrent pneumothorax [7–9]. Several published reports suggest thoracoscopic pulmonary resections, including segmentectomy and lobectomy is safe, with the potential advantage of shorter hospital stay, quicker recovery, and better cosmetic result treatment modality [10–12]. VATS is being used extensively for evaluation and treatment of mediastinal masses. It provides a better visualization of the tumor and its anatomic connections of mediastinal structures. VATS thymectomy and removal of neurogenic mediastinal tumors in children produced good results with minimal complications [13, 14]. Other advanced intrathoracic procedures such as repair of congenital diaphragmatic hernia and eventration, primary repair of esophageal atresia and tracheoesophageal fistula (TEF), and closure of patent ductus arteriosus ligation have been described in children.

Some surgical interventions have essentially been performed with minimally invasive techniques over the past several years, such as single primary pull-through procedure for Hirschsprung's disease and pull-through procedure for high anorectal malformations and fundoplication of gastroesophageal reflux. The early results of these techniques are encouraging and more follow-up studies are required to determine and confirm their advantages. However, it has been reported that eighty percent of pediatric surgeons in USA have performed at least fifty different kinds of laparoscopic procedures [15]. Even fetoscopic intervention has been demonstrated to correct major congenital malformation such as fetal urethral obstruction or complications associated with monochorionic twins. As new technologies keep on evolving, robot-assisted laparoscopic surgery such as in the Da Vinci Surgical System keeps on pushing the boundaries of both diagnostic and therapeutic laparoscopy into the next generation.

Minimally invasive surgery in pediatric populations is increasingly being performed because it offers advantages over conventional surgery such as less tissue injury with less blood loss, decreased need for pain medication, decreased scar formation, shorter hospital stays, and faster recovery. 

## 2. First Steps of Minimal Invasive Surgery in Taiwan 

Europe has been the heart of development for minimally invasive techniques. Minimally invasive techniques have also now been well developed, established, and standardized in USA in recent years. The application and development of minimally invasive techniques in pediatric patients may have been lagging in Taiwan. Up until the middle of the 1990s, the surgical community in Taiwan was still not well prepared to accept the coming era of minimally invasive surgery. Fortunately, more recently there have been many surgeons entering this field with a basic conventional surgical background. In the beginning, laparoscopy was performed in only a few specialties such as gynecology, urology, and general surgery. In addition, only relatively short and simple surgeries were considered. For example, diagnostic laparoscopy for the purpose of evaluating contralateral patent processus vaginalis was reported in the early 1990s, but the first case has been definitely shown in Taiwan until 1995, reported by Chin et al. [16, 17]. They reported the use of intraoperative laparoscopic examination in patients receiving unilateral herniorrhaphy [18]. Intraoperative nonpuncture laparoscopic examination using the hernia sac or hydrocele incision wound as the scope entrance has been successfully conducted in children with unilateral inguinal hernia or hydrocele [15]. Furthermore, contralateral patent processus vaginalis can be visualized and repaired simultaneously with this method. In addition, Wu and Hsu first published procedural laparoscopy report in 2002. In his report, the laparoscopic Ladd's procedure was performed safely in the three pediatric patients with intestinal malrotation who enrolled in his study [19]. The potential of endoscopic surgery continues to be explored in Taiwan in the recent years. However, the low birth rate has led to a reduction in the patient population in need of pediatric surgeries. According to the Population Reference Bureau's annual report in 2011, Taiwan has the lowest birthrate in the world, with only one baby born per woman. As a result, pediatric surgeons in Taiwan will face a decline in opportunities to accumulate surgical experience. Even in face of such difficulties, minimally invasive techniques in Taiwan have remained stable in ongoing development and progress, especially in Southern Taiwan, under the leadership of experienced surgeons. 

Take the authors' hospital, for example, which is a medical center in southern Taiwan. Pediatric minimally invasive techniques have been in use since April 2002, and more than 1000 cases of thoracoscopic and laparoscopic surgeries have been completed. The age distribution ranged from 36 hours from birth to 18 years of age. The number of cases continues to grow since, including six cases in 2002, 50 cases in 2005, 75 cases in 2006, 152 cases in 2007, and 154 cases in 2008. Minimally invasive surgery in 2008 accounted for 34.8% of the total patients undergoing surgeries. Compared with previous years, the postoperative complications have dropped significantly with reduction in pain control medication and increase in patient satisfaction. Currently, laparoscopic appendectomy and laparoscopic hernia repair have become routine surgical approaches in our department. Laparoscopic treatment accounted for 94.6% of all acute appendicitis, and only nine cases adopted traditional surgery. In addition, there have been over 100 cases of laparoscopic hernia repair. 

We adopted the laparoscopic treatment for a 16-year-old adolescent with complicated blunt hepatic injury in 2006, which was the world's first successful laparoscopic treatment of acute liver trauma. In 2007, we were successful with laparoscopic treatment of a six-year-old girl with a giant retroperitoneal T-shaped duplication of descending colon. In 2008, we reported laparoscopic treatment for recurrent episodes of intussusception and inguinal hernia [20]. The majority of general surgeries can be performed by laparoscopic techniques; it has replaced open surgery as the diagnostic and therapeutic gold standard for some complicated problems. In the future, the potential for endoscopic surgical procedures should continue to be explored. 

## 3. Minimal Pediatric Thoracic Surgery in Taiwan

### 3.1. Pectus Excavatum: Nuss Procedure

Pectus excavatum (PE) is a common chest wall malformation thought to be caused by the excessive growth of the costal cartilage, which produces a concave anterior chest wall [21, 22]. Children with PE appear to have an age- and height-corrected total lung volume (TLV) equivalent to that of their healthy counterparts. The data also show no evidence of deterioration in lung volume development in PE patients as they grow [23]. In 1998, Nuss et al. introduced a minimally invasive technique for repairing PE [24]. This procedure involves the insertion of a prebent metal bar to elevate the depressed sternum. After the procedure, there is a definite but minimal increase of intrathoracic volumes which might be beneficial to patients' pulmonary function [25].

However, little is known regarding stress and strain on the deformed ribs after inserting the bar. On sonography, it has been proven to reveal more fractures than when first anticipated on radiography. Therefore, the use of ultrasonography is appropriate for identifying cartilage angulation and bony fracture after the Nuss procedure [26]. Postoperatively, all patients showed various degrees of deformation from the second to sixth cartilages bilaterally. Nearly one-third of patients had significant changes, including acute angulation of the costochondral junction and rib fractures near the pectus bar, especially in those who were older than 10 years or had a high degree of sternal elevation [27].

Finite element analysis (FEA) model was specifically used to analyze the stress and the strain distributions induced in the chest wall by a Nuss procedure. By this analysis, the simulation results indicated that greatest strain occurred at the third through seventh cartilages, especially where they join the sternum and ribs. A high bilateral stress distribution was also found over the backs of the third to the seventh ribs near the vertebral column [28].

The Nuss procedure involves making incisions on each side of the chest and creating a skin tunnel from the lateral thoracic incision to the top of the pectus ridge on each side. Traditionally, the length of the pectus bar is determined by measuring the distance between the midaxillary lines with a measuring tape and subtracting 2.5 cm. The success of the Nuss procedure is based on a prebent bar shaped to the desired curvature of the chest wall, and critically depends on the length and curvature of the bar. By computed tomography measurement, the length of the bar can be measured precisely and can be shaped perfectly to match the chest wall contour. Most importantly, the sternum can be elevated to achieve the desired CT index. Up to 16% of the patients undergoing the Nuss procedure may have errors in bar measurement, especially older patients (>18 years), females with developed breasts, and patients with unusually high sternal angulation. Computed tomography offers more precise bar length determination and better bar design, compared with the traditional method [29].

The common complications associated with the Nuss procedure are pneumothorax, pleural effusion, postoperative pain, wound infection, and bar displacement. The postoperative complication of hemothorax associated with the Nuss procedure is rare and the risk is less than 1% in recent reports. Most of the hemothorax occurs in the first 24 h after surgery. Thoracoscopy may be performed to find the source of the bleeding from the mediastinum, intercostal, and internal mammary vessels. Trauma and exercise may increase the friction between the bars and ribs and become the risk factor for the late-onset hemothorax after the Nuss procedure. In a rare case in Taiwan [30], the late-onset bilateral hemothorax might have resulted from the friction between the implants and internal mammary vessels, causing the life-threatening hemothorax with hypovolemic shock.

### 3.2. Primary Spontaneous Hemopneumothorax

Primary spontaneous hemopneumothorax (PSHP) is infrequent and accounts for 3.1% of all cases of primary spontaneous pneumothorax (PSP) [31]. Primary spontaneous hemopneumothorax is more common in thin men with a mean age ranging from 22.1 to 34.0 years, and most reports are of Asian origin. In a review of 136 patients with PSHP, in most cases, the bleeding sources are identifiable; bleeding ariss most commonly from torn pleural adhesions (59.5%), ruptured well-vascularized bullae (18.3%), or torn congenital aberrant vessels between the parietal pleura and bulla (16.9%) [32–34].

Primary spontaneous hemopneumothorax (PSHP) is a rare but potentially life-threatening condition with two cardinal symptoms, chest pain, and dyspnea. Physical examination usually reveals the immobile, ballooned hemithorax with hyperresonant percussion and diminished breath sounds. Distant amphoric respiration may be heard, accompanied by whispered pectoriloquy.

However, several cardiopulmonary indications in children can certainly mimic primary abdominal diseases, ranging from the mostly seen pneumonia, pleuritis, myocarditis, and pericarditis, to the lesser seen acute mediastinitis, aortitis, and spontaneous rupture of the esophagus. Chen et al. [35] proposed that Primary spontaneous hemopneumothorax simulates acute abdominal affections without the cardinal symptoms, chest pain and dyspnea. The explanation for the abdominal pain in PSHP is attributed to the common innervation of the lower parietal and diaphragmatic pleura and the abdominal viscera. Abdominal rigidity is probably produced by basal pleurisy. Besides the simulation of gallbladder disease [36], SHP may also simulate appendicitis, bleeding peptic ulcer, or even perforated peptic ulcer.

The initial treatment of a large-caliber chest tube may help to evacuate blood, reduce the risk of a clotted hemothorax, and provide a method for continuous monitoring of blood loss. Some studies have concluded that conservative treatment is adequate if bleeding persists less than 24 hours after chest tube insertion [37]. However, if the patients with PSHP are initially managed nonoperatively, most of them ultimately undergo surgery for one of the following conditions: refractory clinical deterioration (profuse blood loss), reappearance of an unstable condition (rebleeding), and later complications (reactive fluid collection, clot empyema, or persistent air leak) [38]. Recent studies have been concerned with the importance of early and aggressive treatment of PSHP in the stable condition [31, 38]. Early operation avoids unnecessary excessive bleeding, and the procedure could be performed in a leisurely and attentive manner because these patients were more likely to be stable after resuscitation [38].

Standard thoracotomy remains the standard method of management for complicated cases such as PSHP with shock or concurrent contralateral pneumothorax. However, currently, the role of VATS is evolving for hemodynamically stable patients in the acute phase, such as for diagnosis or bleeding control [37]. VATS is an acceptable approach for the treatment of PSHP and the early, prompt VATS is not inferior to the conventional thoracotomy [38]. There are a number of reasons for this. Firstly, patients with PSHP are mostly younger and are in relatively stable condition after timely resuscitation and tube thoracostomy. Secondly, VATS offers easier access to bleeders near the Sibson fascia or the roof of the pleural cavity with the use of angled telescopes and high-resolution microcameras.

In conclusion, VATS is a feasible option in treating patients with PSHP and concurrent contralateral pneumothorax after adequate resuscitation and drainage of the contralateral pneumothorax, even when massive hemothorax or hypotension is present [39].

### 3.3. Hybrid Combination of Small Subxiphoid Incision and Thoracoscopic Thymectomy for Juvenile Myasthenia Gravis

Patients with myasthenia gravis who received thymectomy achieved complete stable remission and pharmacologic remission at a significantly higher rate than patients who were treated with nonsurgical procedures [40, 41]. In Juvenile Myasthenia gravis, there is a congenital variety and also a hereditary variety of the condition, but the most frequent cause is an immune-mediated neuromuscular blockade, and the disease is generally nonhereditary. There has been some reluctance in offering thymectomy as a treatment modality to children considering the potential of compromising the long-term immunity in them. However, in various studies, where thymectomy has been done in children, there has not been any adverse effect reported [13]

Thymectomy as a therapeutic modality for MG has been practiced since 1911. Till recent years, the approach to the thymus was either transcervical or transsternal. Although the former was technically more challenging, the trans-sternal route offered a possibility of more complete removal of thymic tissue and mediastinal clearance, but this was at the expense of a significant morbidity of the procedure and thus had much lesser patient compliance. Video-assisted thoracic thymectomy has been used to treat thymus disease since 1992, and several standard surgical procedures have been shown to result in a favorable outcome [40, 42, 43]. Although some of the reports have suggested the need to perform a bilateral VATS, this suggestion has not yet been consistent in all studies. There have also been some suggestions to approach the thymus from the left chest, but others have highlighted the problems of the arch of aorta and difficulty in accessing the brachiocephalic vein through this approach and have preferred the right chest approach [13].

A hybrid combination of small subxiphoid incision and thoracoscopic thymectomy, first described in 2002, provides an excellent visualization of the bilateral pleural cavities and a more radical thymectomy than a right-side thoracoscopic approach [44, 45]. 

Compared with unilateral thoracoscopic approach, the hybrid combination technique with the aid of sternal lifting provides easy identification and control of superior vena cava, the innominate vein, thymic vessels, and thymic horns. In addition, advantages of bilateral thoracoscopic approach are complete and safe dissection of perithymic fat along the bilateral phrenic nerve. Yeh et al. [46] proposed that postoperative recovery is better in endoscopic surgery, including wound pain and cosmetics. The endotracheal tubes were all left for one day to prevent postoperative respiratory distress; no morbidity or mortality was noted. One had complete remission 2 years later (Osserman class IIb), and three had improvement in symptoms and medication, according to Yeh's article.

Therefore, thoracoscopic thymectomy has been successfully applied in pediatric surgery (e.g., myasthenia gravis and thymoma) and can be performed in patients as young as 1.6 years.

### 3.4. Pediatric Empyema

In infants and children, empyema is usually associated with pneumonia. Posttraumatic empyema is a rare complication of trauma with an incidence of 1.6–2.4% in trauma patients [47]. In the early stage, antibiotics and chest tube drainage may be sufficient. Traditionally, treatment of the exudative stage is performed with tube thoracostomy when the pleural fluid is still thin. As the disease progresses to the fibrinopurulent and even the organized stage, surgical intervention is often needed. The diagnosis is usually confirmed by the appearance of pleural fluid in radiology, including plain film, sonography, and CT scans. However, the CT characteristics of parapneumonic effusions do not allow radiologists to accurately predict empyema. Furthermore, the accurate staging of empyema is difficult. The exudative stage can be as short as 24 h. The fibrinopurulent stage occurs within 2–10 days. The late stage can occur within 2–4 weeks. 

As we know, the selection of proper therapy depends on the stage of empyema. The treatment decision is always made with the combination of image studies, the patient's medical history, and clinical symptoms. In any case, the goal of therapy is to eliminate the empyema, control infection, reexpand the trapped lung, and return respiratory function to normal as soon as possible. Since the late 1990s, video-assisted thoracoscopic surgery (VATS), which causes less trauma and better cosmetic outcomes, has become a good alternative of surgical intervention. 

During the VATS, the two-and one-lung ventilations are required. One-lung ventilation is indicated when isolation of one lung is necessary to prevent contamination by the other lung or flooded with the contents of the abscess cavity during manipulation of the diseased lung [48, 49]. For adults, one-lung ventilation is achieved most commonly with a double-lumen tube or a Univent tube. A wire-guided endobronchial blockade and single-lumen endobronchial tube with tube exchanger are used currently for lung separation in the pediatric population with the same lung deflation, efficacy, and safety [48, 49].

Fibrinolytic therapy using urokinase has recently been introduced [50–52], but it requires 2 or more weeks to complete. Moreover, the failure rate is high. Using intrapleural streptokinase washing for fibrinopurulent pediatric empyema yielded comparable success rates to the operative approach, with less morbidity [53]. But the VATS for pediatric empyema, lung tissue, must be preserved as much as possible. The regenerative ability of children is great, and tissue can only be resected under very careful decision making.

Primary VATS has been studied in several series, which all concluded that VATS is a safe, effective, and a first-line therapeutic choice for pediatric empyema, and also results in shorter hospital stay, shorter period of chest tube drainage, less blood transfusion, and less radiation exposure. Moreover, VATS can be a safe and effective treatment for neonatal empyema [54]. However, if patients have the below factors necrotizing pneumonia, requiring preoperative intensive care unit admission or undergoing preoperative chest tube drainage, it means that they are at high risk for developing complications and requiring longer hospital stay after VATS [35].
